# New Methods Section in *PLOS Computational Biology*

**DOI:** 10.1371/journal.pcbi.1002972

**Published:** 2013-03-07

**Authors:** Thomas Lengauer, Ruth Nussinov

**Affiliations:** 1 Max Planck Institute for Informatics, Saarbrücken, Germany; 2 Basic Science Program, SAIC-Frederick, Inc., Center for Cancer Research Nanobiology Program, Frederick National Laboratory for Cancer Research, National Cancer Institute, Frederick, Maryland, United States of America; 3 Sackler Institute of Molecular Medicine, Department of Human Genetics and Molecular Medicine, Sackler School of Medicine, Tel Aviv University, Tel Aviv, Israel

*PLOS Computational Biology* is the Public Library of Science journal that targets new biology that is facilitated by computational methods. Since the inception of the journal, biology has been at the center of the scope of *PLOS Computational Biology*. Thus, each submission is required to put advances in biology into its focus in a very concrete manner and not only focus on computation. This is in contrast to other, more technically oriented journals in the area of computational biology.

The new Methods section of the journal acknowledges the fact that a major methodical advance can, in itself, be so relevant that it deserves transcending the technological domain and being presented to a broader readership including not only computational biologists, but also biologists, the targeted readership of this journal. For this reason, *PLOS Computational Biology* has installed a special type of submission, the Methods paper. As the scope statement of the journal spells out, “Methods papers should describe outstanding methods of exceptional importance that have been shown, or have the promise to provide new biological insights. The method must already be widely adopted, or have the promise of wide adoption by a broad community of users. Enhancements to existing published methods will only be considered if those enhancements bring exceptional new capabilities.”

In order to render the processing of Methods papers as effective as possible and to limit the effort required on the part of authors and reviewers, a presubmission inquiry is mandatory for Methods papers. In such an inquiry, the authors are requested to present a concise abstract-like statement on what the manuscript they plan to submit entails and why the authors feel that it fits the Methods section of the journal. Presubmission inquiries are given top priority in the paper handling process; the median processing time should be about a week. Within that time, submission is either encouraged or discouraged.

The Methods section of the journal is handled by Deputy Editor Thomas Lengauer.

